# Expanding the mutational and clinical spectrum of Chinese intellectual disability patients with two novel *CTCF* variants

**DOI:** 10.3389/fped.2023.1195862

**Published:** 2023-08-17

**Authors:** Bo Tan, Sihan Liu, Xiaoshu Feng, Xin Pan, Guanhua Qian, Li Liu, Xu Zhang, Hong Yao, Xiaojing Dong

**Affiliations:** ^1^ Department of Gynecology and Obstetrics, The Second Affiliated Hospital of Chongqing Medical University, Chongqing, China; ^2^ Institute of Rare Diseases, West China Hospital of Sichuan University, Chengdu, China

**Keywords:** RNA-seq, novel variant, *CTCF*, intellectual disability, clinical diagnosis

## Abstract

CCCTC-Binding Factor (*CTCF*) is a protein-coding gene involved in transcriptional regulation, insulator activity, and regulation of chromatin structure, and is closely associated with intellectual developmental disorders. In this study, we report two unrelated Chinese patients with intellectual disability (ID). According to variant interpretation results from exome sequencing data and RNA-seq data, we present two novel heterozygous *CTCF* variants, NM_006565.3:c.1519_2184del (p. Glu507_Arg727delins47) and NM_006565.3:c.1838_1852del (p.Glu613_Pro617del), found in two distinct unrelated patients, respectively. Moreover, RNA-seq data of patient 1 indicated the absence of the mutant transcript, while in patient 2, the RNA-seq data revealed a *CTCF* mRNA transcript with a deletion of 15 nucleotides. Notably, the RNA sequencing data revealed 507 differentially expressed genes shared between these two patients. Specifically, among them, 194 were down-regulated, and 313 were up-regulated, primarily involved in gene regulation and cellular response. Our study expands the genetic and clinical spectrum of *CTCF* and advances our understanding of the pathogenesis of *CTCF in vivo*.

## Introduction

Intellectual disability (ID) is a neurodevelopmental disorder characterized by deficits in intelligence and adaptive functioning of varying severity before the age of 18. The global prevalence of ID is around 1%–3% in the general population and approximately 1% in China. ID can manifest as either an independent feature known as non-syndromic ID (NSID), or it can be accompanied by facial dysmorphic features and other morphological anomalies. Indeed, the majority of ID cases are attributed to genetic abnormalities. With the development of next-generation sequencing, researchers have identified more than 1,000 genes causally linked to ID (1–3).

*CTCF* (CCCTC-Binding Factor) is a protein-coding gene (OMIM: 604167) involved in a variety of chromatin regulatory processes, including gene expression, chromatin higher-order organization, and maintenance of chromatin 3D structure (4, 5). The *CTCF* gene consists of 12 exons that encode eleven zinc finger (ZF) domains. Numerous studies have identified to date 59 pathogenic variants in the *CTCF* gene associated with autosomal dominant ID, including 5 large deletion variants (1, 6–13). It is worth noting that most of these variants are *de novo*, meaning that they are newly occurring in the affected individuals and result in the loss-of-function of one copy of the *CTCF* gene. The association of *CTCF* with ID was first reported in 2013 by Gregor et al., who identified two *de novo* frameshift pathogenic variants, c.375dupT (p.Val126Cysfs14) and c.1186dupA (p.Arg396Lysfs13), as well as one *de novo* missense pathogenic variant, c.1699C>T (p.Arg567Trp), in *CTCF* in four syndromic ID patients (1). Subsequently, several studies reported numerous pathogenic *CTCF* variants in patients with ID and neurodevelopmental disorders (12, 14). While only three *de novo CTCF* disease-causing variants (c.615_618delGAAA[p.Lys206Profs15], c.1699C>T[p.Arg567Trp], c.329dupT[p.Gly111fs29) have been identified in Chinese patients associated with the neurodevelopmental disorder, there is an urgent need to report additional genetic and clinical features from Chinese ID patients to expand the spectrum of *CTCF* variation in the Chinese population (11).

Here, we present the genetic and clinical characterization of two unrelated Chinese ID patients with novel *CTCF* variants (NM_006565.3:c.1519_2184del (p. Glu507_Arg727delins47) and NM_006565.3:c.1838_1852del(p.Glu613_Pro617del). Additionally, we assess the disease-causing mechanism in our patients through RNA sequencing. This study expands current knowledge of the genetic and clinical spectrum of *CTCF* variants and reveals consistent phenotypes and disease mechanisms across populations.

## Materials and methods

### Subjects and clinical information

Patients were recruited from the Second Affiliated Hospital of Chongqing Medical University in Chongqing, China. The patients were clinically assessed, and medical experts and geneticists confirmed no other linked anomalies. This study was approved by the ethical committee of the Second Affiliated Hospital of Chongqing Medical University. Written informed consent was obtained from the patients' parents (approval number: 2023/458).

### Exome sequencing

Genomic DNA from the patients was extracted from peripheral blood collected in EDTA tubes. Exome sequencing was performed on the patients using the Agilent (Santa Clara, CA) version 6 enrichment kit and the Illumina HiSeq 4000 sequencing system (paired-end reads, 2 × 150 bp).

### Variants identification and interpretation

Trimmomatic was used to remove adapter contamination and trim low-quality reads to obtain clean reads (15). Then, the cleaned reads were aligned to the human reference genome (hg19) using the Burrows–Wheeler Alignment tool (16). DNA variants were called following the Genome Analysis Toolkit software best practices (17). Then, variants were annotated using Variant Effect Predictor (VEP) (18–22). Multiple computational predictive tools were applied to predict the pathogenicity of the detected variants (23–29). Further, variants with an allele frequency (AF) of <0.1% were retained for downstream analysis. According to the ACMG/AMP guidelines, all retained variants were classified into pathogenic (P), likely pathogenic (LP), variants with unknown clinical significance (VUS), likely benign (LB), or benign (B) (30). The putative diagnostic variants were experimentally validated by Sanger sequencing (ABI 3730xl Genetic Analyzer) and real-time PCR.

### Copy number variants detection and interpretation

Copy number variations (CNV) were called using ExomeDepth (31) with default parameters, utilizing the BAM files generated from earlier. A set of 10 samples from our in-house control data served as the reference set. When examining potential CNV calls on the X chromosome, only samples of the same sex were considered for correlation. CNVs with a Bayes factor (BF) value below 50 were filtered out. Subsequently, each CNV call underwent additional annotation using the VEP, and classified according to the guidelines proposed by ACMG and ClinGen for CNV interpretation (32).

To validate the presence of CNVs, we performed CNV-seq analysis following the protocol proposed by Dong Zirui et al. (33). Briefly, mapped reads were grouped into 5 kb bins based on their mapped positions (hg19). The coverage of each bin was calculated using the mapped read depth within the bin and underwent a two-step bias correction (GC correction and population-level normalization). A CNV was considered a deletion if its average copy ratio was less than 0.6 or a duplication if it was greater than 1.4. Additionally, quantitative PCR (qPCR) was employed as an additional validation technique. The qPCR reactions were conducted using the SYBR Green I PCR Master Mix (Applied Biosystems) on the Applied Biosystems 7500 Fast Instrument.

### RNA sequencing

Total RNA was extracted from the peripheral blood of these two patients and enriched by oligo-dT bead capture, separately. cDNA was synthesized according to the manufacturer's protocol, and cDNA libraries were constructed using the Illumina TruSeq stranded mRNA sample prep kit protocol (Illumina). Pooled samples were sequenced using a NovaSeq 6000 sequencing system.

### Differential expression analysis

The clean RNA-sequencing reads were mapped to the human reference genome (hg 38) using STAR (2.7.8a) with the Gencode v29 annotation (34). Blood samples (*n* = 735) from GTEx v8 data were used as controls (35). Combat was applied to remove the batch effect between our data and GTEx data. Raw read counts were log-transformed by R package VOOM (36) first, filtering those with log2(CPM) < 0 in more than 75% of the samples. Differential expression analysis of the remaining genes was performed using the Limma (37) package while controlling for biological covariates and hidden factors identified by SVA (38). Gene was defined as a differentially expressed gene (DEG) with an adjusted *p*-value <0.05 and |log-transformed fold-change|>1. Pathway enrichment analysis of DEGs was carried out using KOBAS-i (39). The adjusted *p*-value cutoff for significant pathways was set at less than 0.05.

### Validation of alternative splicing isoform

The RNA specimens collected from both the patients and the negative control underwent reverse transcription and subsequent amplification of cDNA using a forward primer specific to *CTCF* Exon 10 (5′-CTGCGGCTTTTGTCTGTTCT-3′) and a reverse primer specific to Exon 13 (5′-CCTCCTCTTCCTCTCCCTCT-3′). The PCR reaction was performed under the following conditions: an initial denaturation at 95°C for 3 min, followed by 40 cycles of denaturation at 95°C for 30 s, annealing at 58°C for 15 s, and extension at 72°C for 45 s. The resulting PCR products were then subjected to Sanger sequencing for further analysis.

### Reverse transcription-quantitative PCR (Rt-qPCR)

Reverse transcription-quantitative PCR (RT-qPCR) was used to measure the relative expression of candidate differentially expressed genes in both patient and control blood cells. The *GAPDH* mRNA was utilized as an internal control, and the experiments were conducted in triplicate. Quantitative PCR (qPCR) analysis was performed using the QuantstudioTM 7 Flex system (Applied Biosystems) with the following amplification program: 95°C for 10 min, 40 cycles at 95°C for 15 s, and 60°C for 1 min.

## Results

### Genetic analysis of patient 1

**Figure 1:**
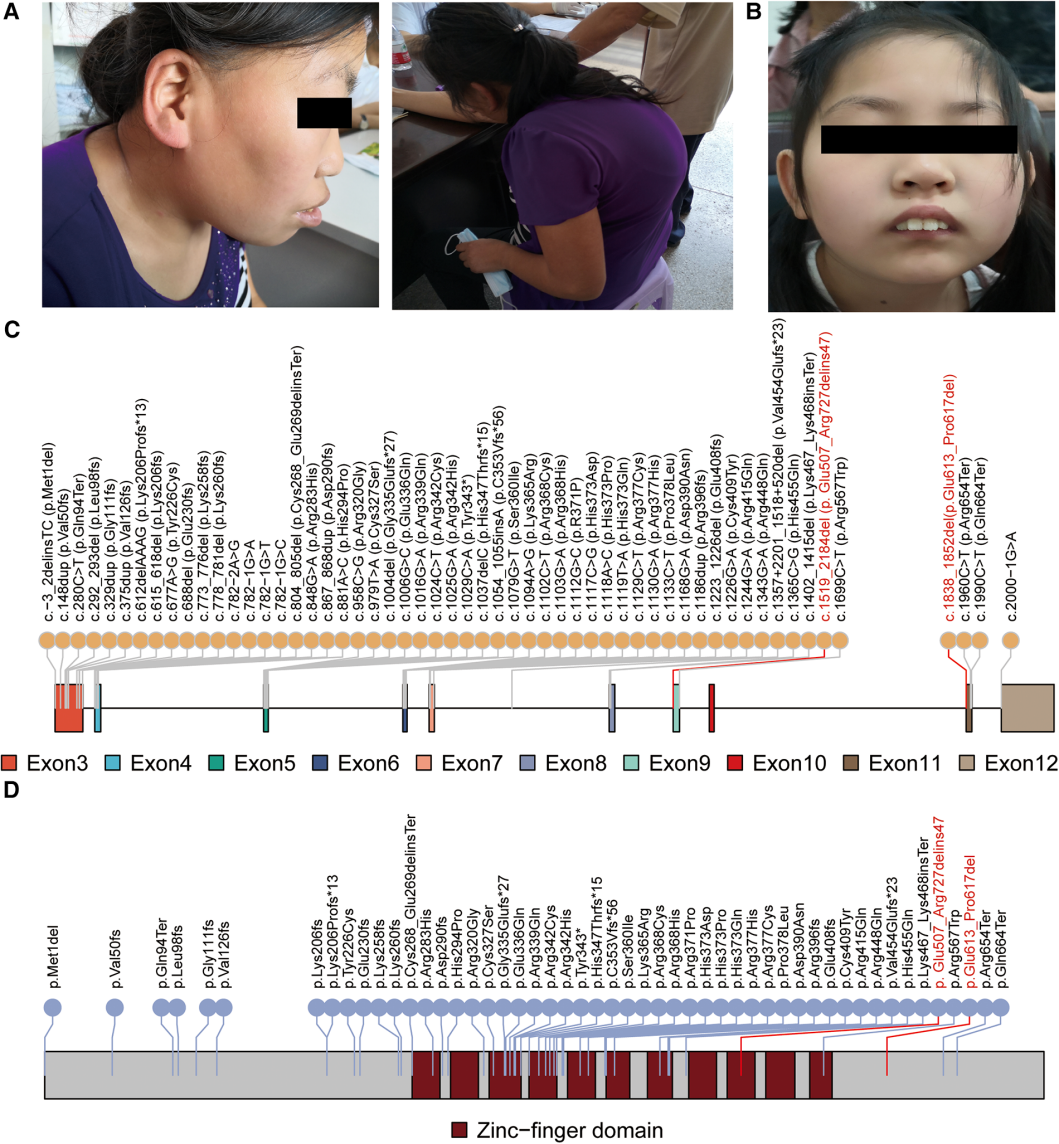
Genetic and clinical features of the patients. The clinical features of patient 1 (**A**) and patient 2 (**B**) are presented. Patient 1 is a 22-year-old female with moderate intellectual disability, strabismus, and scoliosis. Patient 2 is an 11-year-old female with mild intellectual disability, epilepsy, and distinct facial dysmorphic features, including prominent incisors and ptosis. Schematic figures (**C,D**) highlight the positions of the reported pathogenic variants and variants identified in this study (marked in red) within the CTCF mRNA and protein domain, respectively.

**Figure 2:**
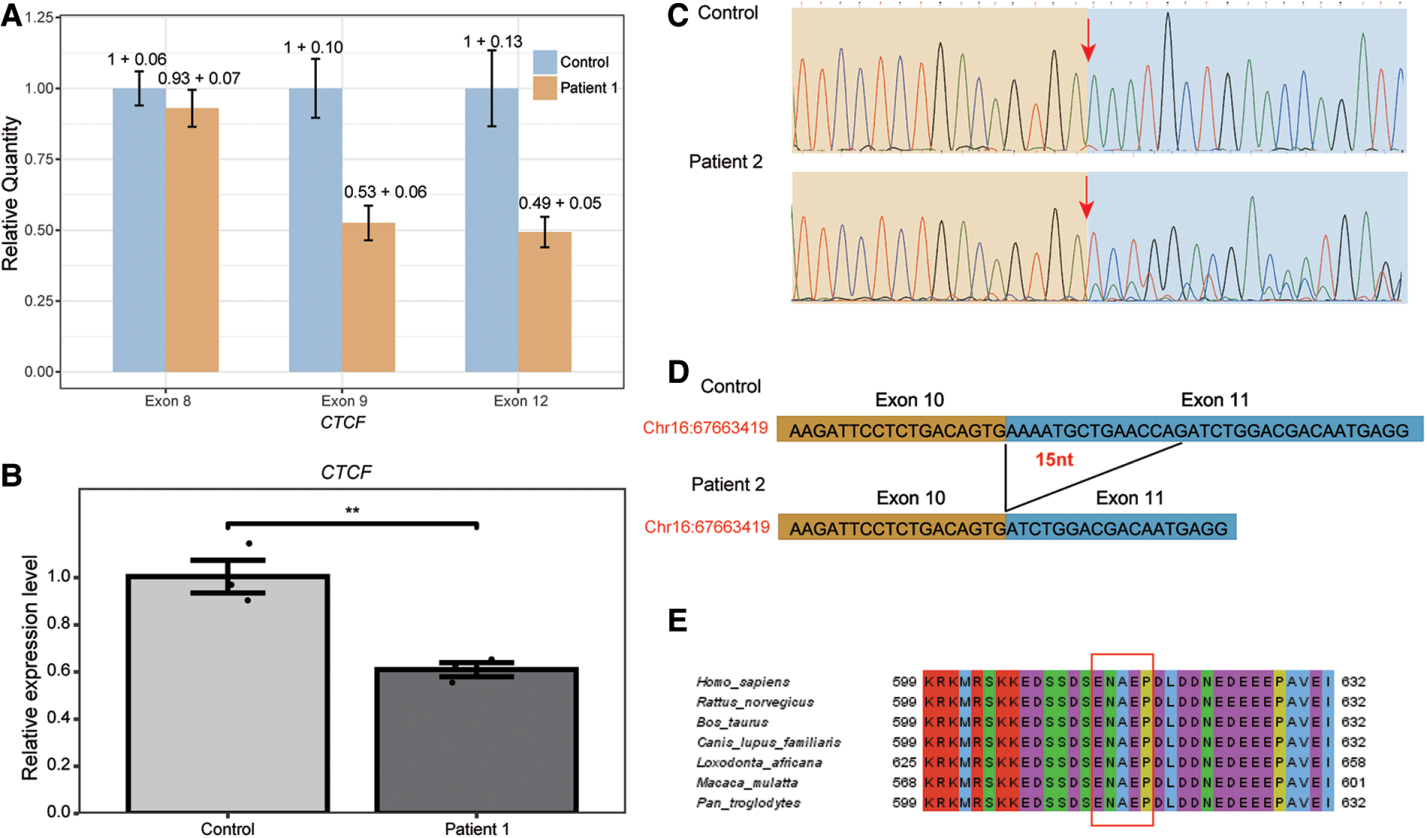
Validation of the two novel heterozygous *CTCF* deletion variants in patient 1 and in patient 2. (**A**) Real-time PCR was performed to validate the deletion of NM_006565.3:c.1519_2184del in the *CTCF* gene of patient 1. The relative quantity and standard deviation of exon 8, exon 9 and exon 12 in patient 1 and control samples was shown. (**B**) The expression of *CTCF* in patient 1 and control blood cells was measured using RT-qPCR, revealing that the *CTCF* expression levels in patient 1 were 50% compared to controls. This significant decrease indicates that the mutant mRNA is undergoing Nonsense-mediated mRNA decay (NMD). Statistical analysis showed a high level of significance relative to controls, with **p < 0.01. (**C**) The RNA specimens from both patient 2 and the negative control were subjected to reverse transcription and cDNA amplification. Sanger sequencing of the resulting PCR products was performed for detailed analysis. Our findings revealed that the *CTCF* c.1838_1852del variant led to a deletion of 15 nucleotides in the *CTCF* mRNA of patient 2. The arrow in the traces highlights the start of the deletion. (**D**) Schematic figures showing alternative splicing events caused by the c.1838_1852del. (**E**) The c.1838_1852del variant results in the loss of five amino acids (red box), which are located within a highly conserved region.

Patient 1 is a 22-year-old female with a height of 146 cm (<3rd percentile) and a weight of 62 kg (89th percentile). She presented with moderate intellectual disability, strabismus, and scoliosis (Figure 1A). Exome sequencing identified a heterozygous 9,503 bp deletion in the genomic region of chr16:67,662,273–67,671,775 (hg19), specifically corresponding to NM_006565.3:c.1519_2184del (p.Glu507_Arg727delins47), in patient 1 (Figures 1C,D). This variant corresponds to a deletion spanning from exon 9 to exon 12 of the *CTCF* gene. As exome sequencing (ES) targets only the coding regions, to assess whether the intronic region of the *CTCF* gene was affected or not, we employed CNV-seq. The CNV-seq data indicated a 16,519 bp deletion in the region of chr16:67,658,826–67,675,344 (hg19), encompassing exon 8 to exon 12 of the *CTCF* gene (Supplementary Figure S1). Confirmation of the presence of the deletion of exon 9 to exon 12 in patient 1 was achieved through qPCR analysis, as illustrated in Figure 2A. Importantly, exon 8 of the *CTCF* gene and the adjacent regions downstream of the *CTCF* gene in patient 1 remained unaffected. This result revealed that the predicted deletion region by CNV-seq is attributed to the deletion of exons 9 to exon 12 and led us to speculate that the breakpoint for the c.1519_2184del variant may reside within the intronic or intergenic regions of the *CTCF* gene. However, the exact breakpoints of the deletion could not be identified. Furthermore, to investigate whether the *CTCF* mRNA in patient 1 underwent nonsense-mediated mRNA decay (NMD), we compared total *CTCF* RNA expression levels to controls. As shown in Figure 2B, the *CTCF* expression levels in patient 1 were 50% compared to controls, indicating that the mutant mRNA is undergoing NMD.

The *CTCF* gene is categorized as a haploinsufficient (HI) gene with a pLI score of 1. The deletion of exon 9 to exon 12 in the *CTCF* gene removes 30% (220/727) of the CTCF protein and could affect the zinc finger domain of the CTCF protein. Based on the ACMG/ClinGen guidelines for CNVs, the NM_006565.3:c.1519_2184del (p.Glu507_Arg727delins47) variant was classified as a likely pathogenic variant [criteria 2D-4 (0.9) = 0.9]. This classification was based on the variant's overlap with established HI genes and its result in exon deletions that include other exons in addition to the last exon, scoring 0.9 according to the guidelines. The inheritance status of this variant remains unknown as parental testing was not possible due to the family relocating, and her parents chose not to participate in the verification process.

### Genetic analysis of patient 2

Patient 2 is an 11-year-old female weighing 32 kg (25th percentile), standing at a height of 135 cm (3rd percentile), with a head circumference of 52 cm (normal percentile). She exhibited mild intellectual disability, epilepsy, and distinctive facial dysmorphic features, including prominent incisors and ptosis. Notably, Patient 2 did not display any characteristic signs of skeletal dysplasia (Figure 1B). Exome sequencing identified a heterozygous splice site variant in the *CTCF* gene (NM_006565.3:c.1838-1_1841del) in patient 2. Sanger sequencing confirmed that the c.1838-1_1841del variant occurred *de novo* (Supplementary Figure S2), as it was not detected in the unaffected parents of patient 2 (PS2_supporting). In addition, this variant has not been annotated in several genomic databases (PM2), including dbSNP and gnomAD, and has not been reported in ClinVar.

To investigate whether the c.1838-1_1841del variant in patient 2 could affect mRNA splicing, we extracted and analyzed RNA from the patient's blood and a control sample using RT-PCR and RNA-sequencing. After separation on a 2% agarose gel, we observed a 516 bp band in both samples. Then, the RT–PCR products were purified and sequenced, and we observed that the c.1838-1_1841del variant caused a deletion of 15 nucleotides in the *CTCF* mRNA, leading to the deletion of 5 amino acids from the CTCF protein in patient 2 (Figures 2C,D). Furthermore, bioinformatics analysis revealed the deletion of five amino acid were located in the highly conserved region of exon 11 of the *CTCF* gene (Figure 2E). As an additional validation of the transcript result attributed to the c.1838-1_1841del variant, the RNA-seq data from patient 2 was visualized using the Gviz R package. Supplementary Figure S3 showcases the specific event that is associated with the c.1838-1_1841del variant. Based on these findings, it was appropriate to update the c.1838-1_1841del variant to c.1838_1852del (p.Glu613_Pro617del), accurately reflecting the 15-nucleotide deletion (Figures 1C,D). Considering the available evidence, the classification of the c.1838_1852del variant as a VUS is justified. Supporting criteria for this classification include PM4 (Protein length changes due to in-frame deletions/insertions), PM2, and PS2_supporting.

### RNA-seq analysis

**Figure 3:**
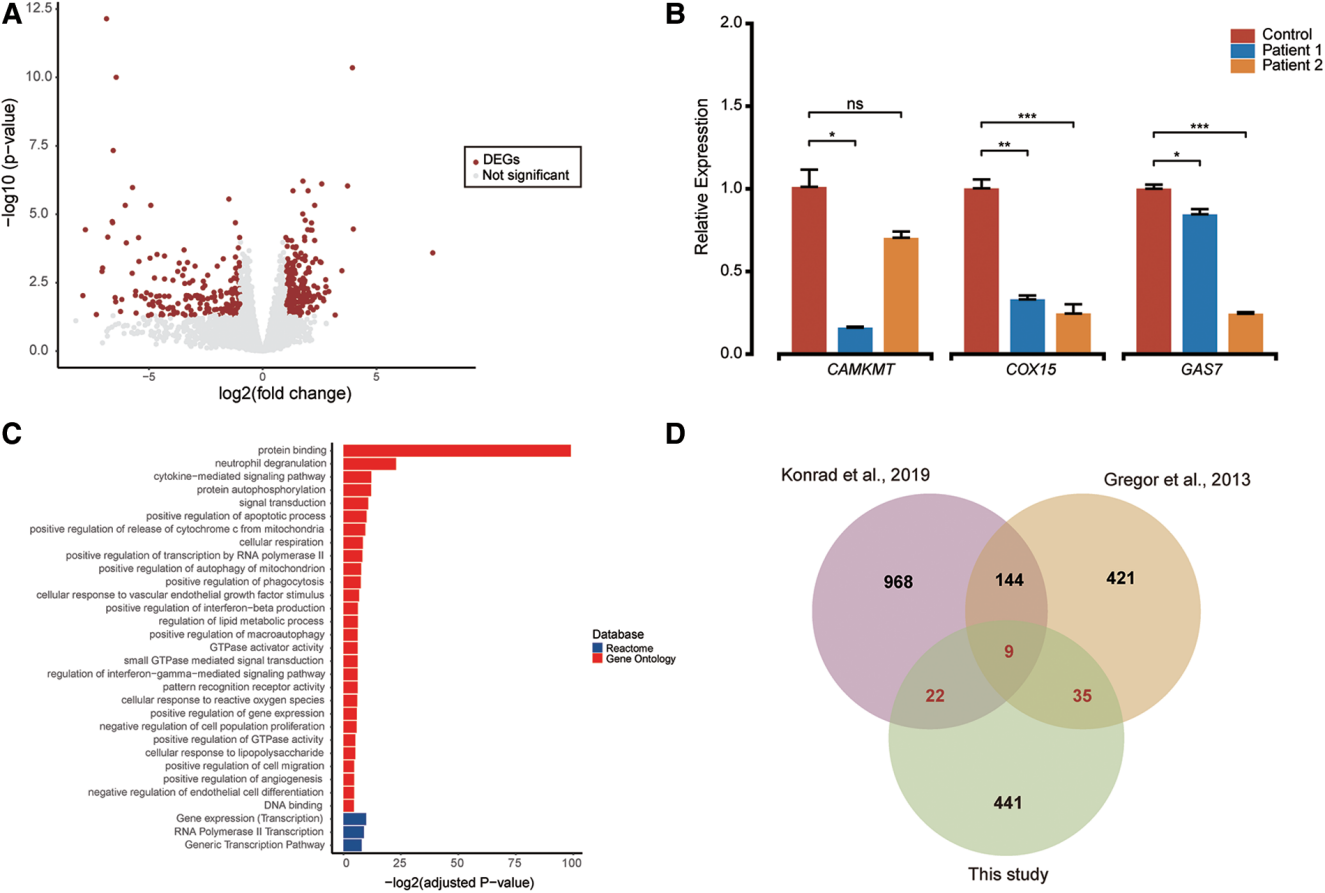
Differential expression analysis and functional enrichment (**A**). (**A**) The volcano plot showcases 507 differentially expressed genes (shown as red dots) out of 14,254 expressed genes, with an adjusted *p*-value < 0.05 and |log-transformed fold-change| > 1 threshold. (**B**) RT-qPCR quantification of candidate genes (*CAMKMT*, *COX15*, and *GAS7*) in patients and control blood cells. Two sample *t*-test was performed to test whether the expression of these genes between cases and control are significant difference or not. Relative normalized expression with standard deviation is presented on the *Y*-axis. Statistical significance is indicated as *p < 0.05, **p < 0.01, and ***p < 0.001. (**C**) Functional enrichment pathways of differentially expressed genes are shown with colors representing pathway items from various databases. The *X*-axis shows the log-transformed adjusted *p* value. Pathways with an adjusted *p* value <0.05 were selected as significant pathways and plotted. (**D**) The Venn plot illustrates the overlap of differentially expressed genes reported in this study compared to findings from two other papers. Differentially expressed genes were defined as genes with an adjusted *p*-value <0.05 and a |log-transformed fold-change| > 1.

RNA-sequencing was conducted on RNA extracted from the blood cells of patient 1 and patient 2, both of whom had pathogenic *CTCF* variants, to elucidate the underlying mechanisms of the disease. A total of 14,254 expressed genes were included in the analysis, and 507 differentially expressed genes were identified with adjusted *p*-value <0.05 and |log-transformed fold-change|>1 threshold (Figure 3A and Supplementary Table S1). Among the 507 DEGs, 194 were down-regulated and 313 were up-regulated consistently and shared between the two patients, including several genes associated with neurodevelopmental disorders such as *CAMKMT*. Relative quantification of a subset of genes (*CAMKMT*, *COX15*, and *GAS7*) using RT-qPCR confirmed the accuracy of differential analysis with RNA-seq data (Figure 3B). Enrichment analyses of the DEGs revealed that they were involved in gene regulation and cellular response (Figure 3C and Supplementary Table S2).

## Discussion

Identifying genetic causes of ID is crucial for understanding the molecular mechanisms underlying clinical features and for improving the management of the patients. In this study, we present two novel heterozygous *CTCF* variants NM_006565.3:c.1519_2184del (p. Glu507_Arg727delins47) and NM_006565.3:c.1838_1852del(p.Glu613_Pro617del) identified in two unrelated Chinese ID patients and validated the effect of these two variants on gene expression and splicing of the *CTCF* gene. However, it is important to note that the *de novo* status of the c.1519_2184del variant cannot be confirmed at this time due to the unavailability of parental testing for the patient. Our findings have not only identified the likely genetic causes of disease in these two patients but have also expanded the spectrum of disease-causing variants that can cause ID.

The *CTCF* gene is definitively associated with autosomal dominant syndromic ID, which was approved by the ClinGen Intellectual Disability and Autism Gene Curation Expert Panel on 7/21/2021 (40). A total of 54 pathogenic SNV/INDEL variants have been documented as causative for ID in the *CTCF* gene (Supplementary Table S3). These variants are primarily concentrated within exon 3 to exon 12 of the *CTCF* gene (Figure 1C) (1, 6–13). Furthermore, the majority of these variants are located in the zinc-finger domain and result in mild to moderate intellectual disability along with additional highly variable phenotypic features (Figure 1D). For instance, a female patient with a *de novo* c.1024C>T, p.Arg342Cys variant in the *CTCF* gene exhibited mild intellectual disability, a flat face, upslanting palpebral fissures, and a broad nose (13). Of note, previous studies have shown that the neurodevelopmental phenotype in individuals with larger deletions encompassing *CTCF* is not markedly different or more severe than in individuals with intragenic missense or likely gene-disruptive variants (12). This observation was consistent with our study, as the reported two patients showed mild or moderate ID. However, due to their rural background, many clinical investigations have not been conducted, and additional information on their early development, milestones, head circumference, cardiac anomalies, magnetic resonance imaging, and electroencephalography results is unavailable.

In our study, we report two novel heterozygous variants, NM_006565.3:c.1519_2184del (p. Glu507_Arg727delins47) and NM_006565.3:c.1838_1852del(p.Glu613_Pro617del), identified in unrelated Chinese patients with ID. The c.1519_2184del variant in patient 1 cause a partial deletion of the zinc-finger domain. We speculate that the breakpoint for the c.1519_2184del variant may reside within the intronic or intergenic regions of the *CTCF* gene since this variant affects exons 9–12 of the *CTCF* gene while leaving the nearby genes “*CARMIL2*” and “P*ARD6A*” unaffected. Patient 2, carrying the c.1838_1852del variant, shares a notable clinical feature of prominent incisors with previously reported ID patients harboring pathogenic inframe variants in the *CTCF* gene (1, 7). We observed a difference in annotation based on the ES and RNA-seq data for the novel *CTCF* variant identified in patient 2. The discrepancy between the expected results based on the genomic annotation and the RNA-seq results in patient 2 might be attributed to alternative splicing or other post-transcriptional processes. The c.1838_1852del variant in the *CTCF* gene leads to a partial deletion of exon 11 in the *CTCF* mRNA. The deletion leads to the loss of 5 amino acids in a highly conserved region but does not impact the zinc-finger domain. Overall, the c. 1838_1852del variant in the *CTCF* gene may contribute to the patient 2's phenotype. Additional research and functional assays may be required to establish a more definitive clinical significance for the c. 1838_1852del variant.

RNA-seq provides an opportunity to investigate the molecular mechanisms underlying a patient's phenotype. In 2013, Gregor et al. used RNA sequencing to study three patients diagnosed with ID who carried pathogenic *CTCF* variants and discovered broad deregulation of genes involved in the cellular response to extracellular stimuli (1). Similarly, Konrad et al. identified 1,143 differentially expressed genes between five individuals with pathogenic *CTCF* variants and eight healthy controls (12). In our study, we also observed broad deregulation of genes (*n* = 507) in the patients. Among these, 66 DEGs were consistent when compared with the findings from the previous two studies. Moreover, nine genes (*PELI1*, *CSF2RB*, *B3GNT5*, *SLC6A6*, *CPD*, *MEGF9*, *SOD2*, *CLEC7A*, *ABHD2*) exhibited consistent differential expression across all three studies (Figure 3D). In addition, qPCR confirmed that many NDD-related genes, such as *CAMKMT*, *COX15*, and *GAS7* involved in neurodevelopment and cancer, were downregulated in the patients (41–43). *CAMKMT* is linked to Hypotonia-Cystinuria Syndrome (OMIM #606407), which manifests as mild to moderate intellectual disability and respiratory chain complex IV deficiency. The deficiency of the *COX15* gene leads to mitochondrial complex IV deficiency (OMIM #220110), presenting with encephalomyopathic features in the neonatal period for some patients, while others experience developmental regression within the first year of life. Additionally, *GAS7* is associated with schizophrenia and plays a role in regulating neuronal migration and morphogenesis. Pathway enrichment analysis showed that the biological functions of these 507 DEGs were related to gene regulation and cellular response. Our study suggests that the main pathogenic mechanism of variants located in the *CTCF* domain is the imbalance of gene expression and regulation, which is consistent with previous research (1, 12).

In conclusion, our study demonstrates that autosomal dominant intellectual disability can be caused by exon deletion (c.1519_2184del) and inframe deletion variants (c.1838_1852del) in the *CTCF* gene. RT-PCR analysis confirmed that the c.1838_1852del variant led to the partial deletion of exon 11 from the *CTCF* mRNA, which may impact the function of the CTCF protein. Our study also adds to the existing knowledge of the genetic and clinical spectrum of *CTCF* variants in Chinese ID patients and highlights the consistent disease mechanisms of ID patients who carry pathogenic variants in the *CTCF* gene across different populations.

## Data Availability

The datasets presented in this study can be found in online repositories. The names of the repository/repositories and accession number(s) can be found below: Genome Sequence Archive (Genomics, Proteomics & Bioinformatics 2021) in National Genomics Data Center (Nucleic Acids Res 2022), China National Center for Bioinformation/Beijing Institute of Genomics, Chinese Academy of Sciences (GSA-Human: HRA005212). Publicly accessible at https://ngdc.cncb.ac.cn/gsa-human.
