# Supplementary material for: Expanding the mutational and clinical spectrum of Chinese intellectual disability patients with two novel CTCF variants
Source: Front Pediatr. 2023 Aug 17;11:1195862. doi: 10.3389/fped.2023.1195862 (PMC10469948; doi:10.3389/fped.2023.1195862)
Supplement: Supplementary file 1 [file Table1.docx]

Supplementary Material

# Supplementary Figures

##
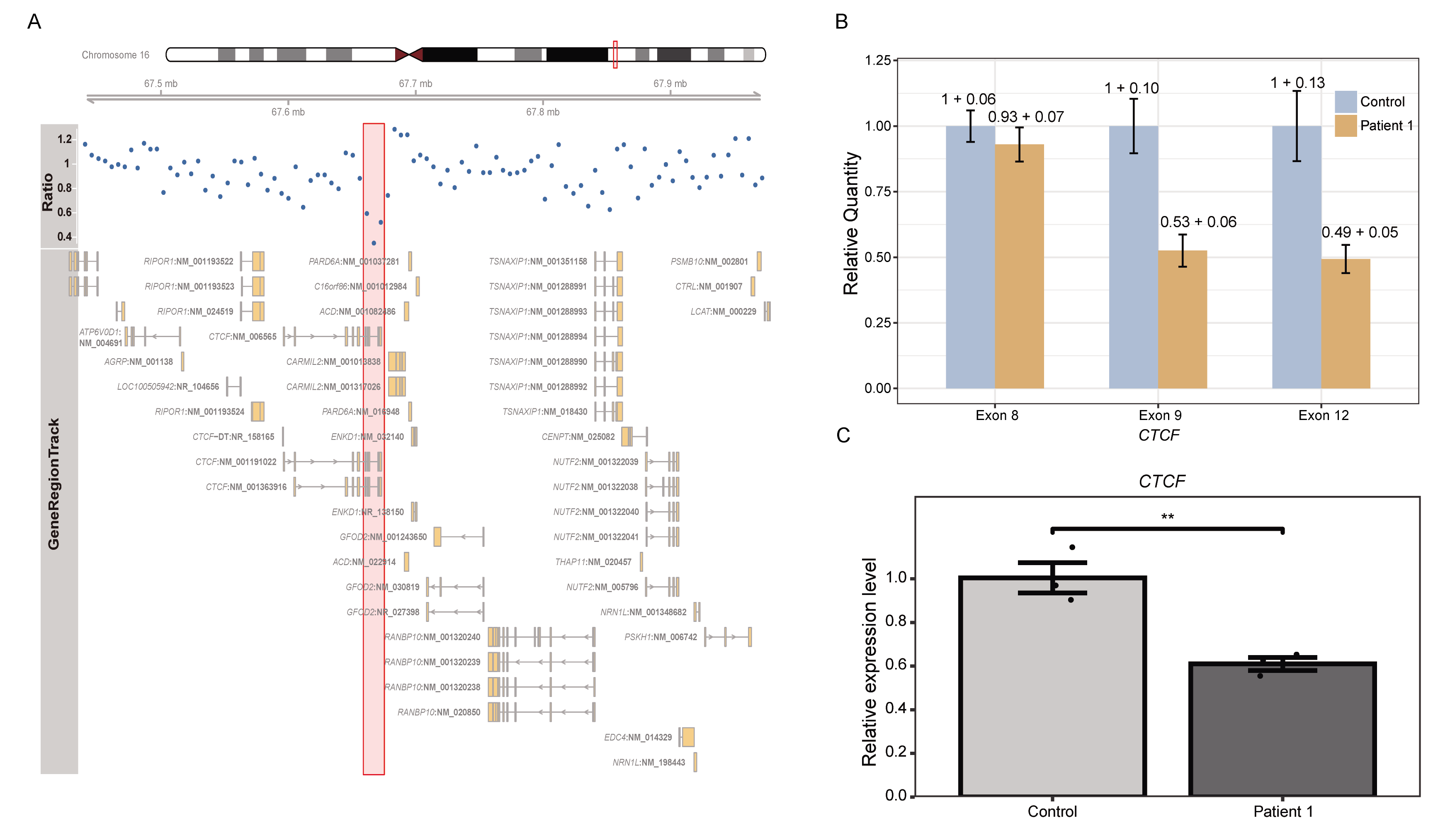


**Supplementary Figure 1:** The copy number ratio of each bin derived from CNV-seq analysis. The bins with a copy number ratio <0.6 are highlighted with a red frame, indicating the presence of deletions.


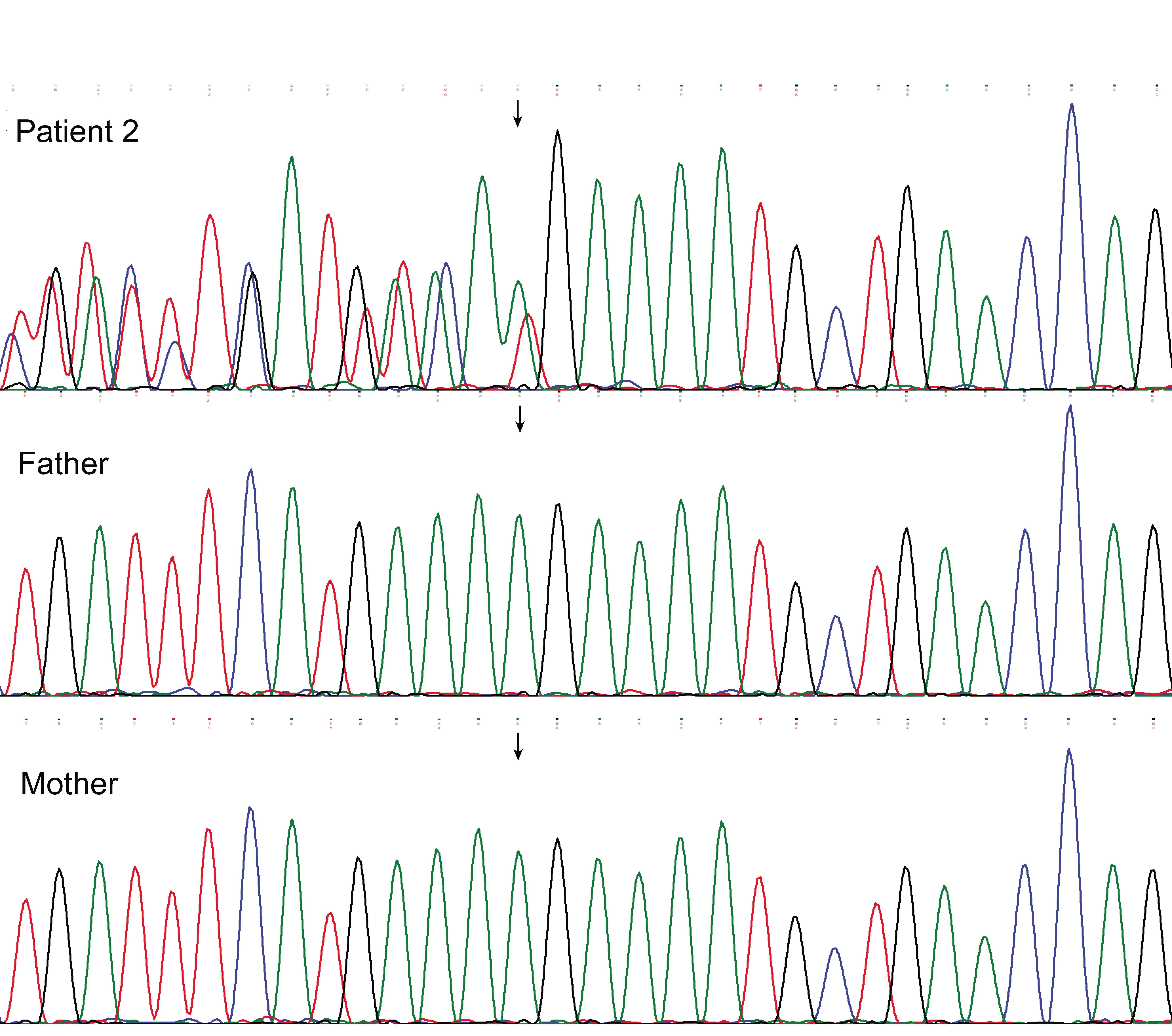


**Supplementary Figure 2:** Results of Sanger sequencing. Sanger sequencing traces of the c.1519_2184del variant in patient 2 and her parents. The arrow indicates the start of the deletion.


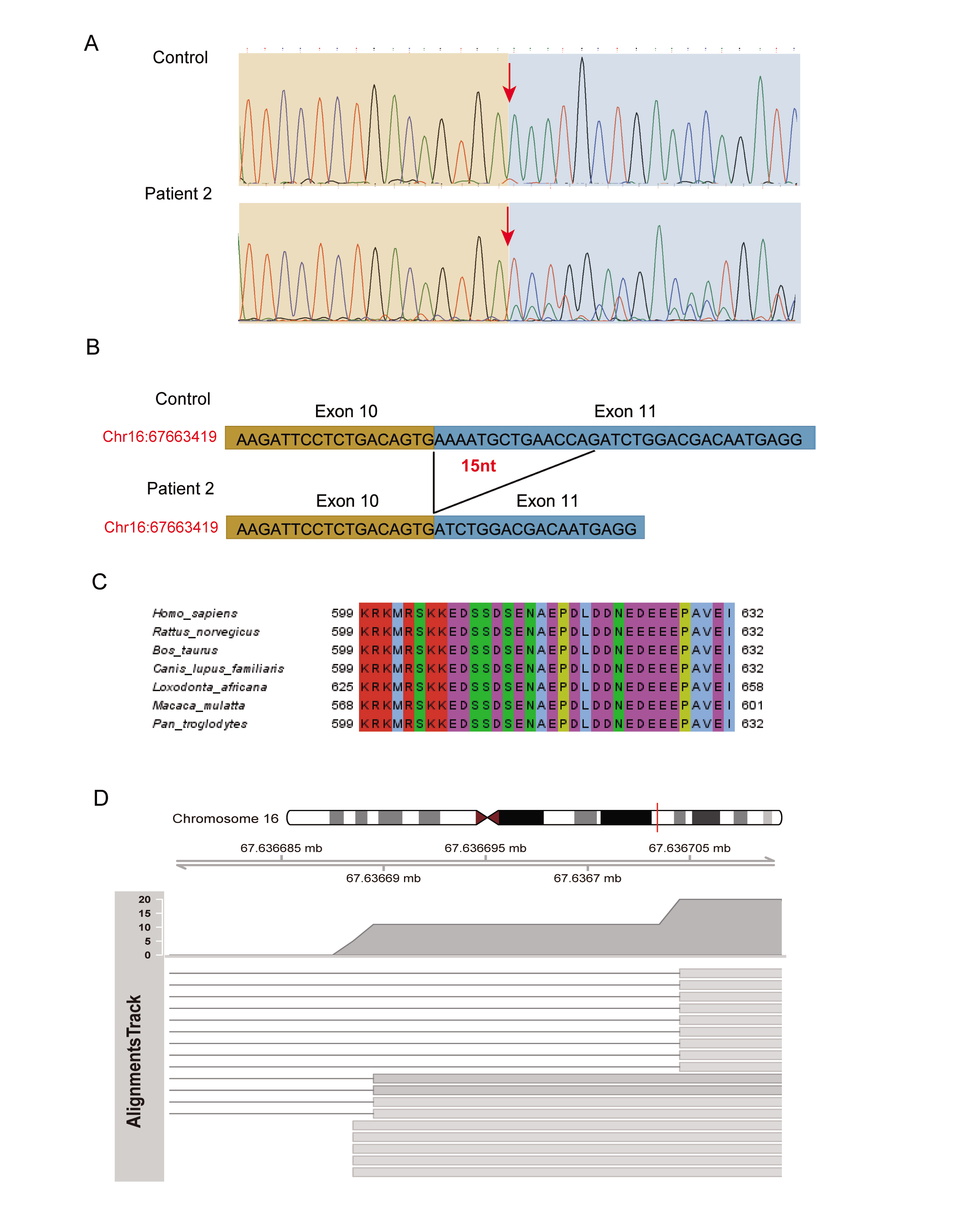


**Supplementary Figure 3:** RNA-seq data of patient 2 visualized using the Gviz R package. The data presented in this figure provides visual confirmation of the specific splicing event associated with the c.1838_1852del variant. The five bars at the bottom represent the duplicate reads in the RNA-seq data. The darker gray bars represent reads where the forward and reverse reads overlap, while the lighter gray bars represent mapped reads.
